# RiCRN1, a Crinkler Effector From the Arbuscular Mycorrhizal Fungus *Rhizophagus irregularis*, Functions in Arbuscule Development

**DOI:** 10.3389/fmicb.2018.02068

**Published:** 2018-09-04

**Authors:** Stefanie Voß, Ruben Betz, Sven Heidt, Nicolas Corradi, Natalia Requena

**Affiliations:** ^1^Molecular Phytopathology, Botanical Institute, Karlsruhe Institute of Technology (KIT), Karlsruhe, Germany; ^2^Department of Biology, Canadian Institute for Advanced Research, University of Ottawa, Ottawa, ON, Canada

**Keywords:** effectors, crinkler (CRN) proteins, arbuscular mycorrhiza, plant symbioses, arbuscule

## Abstract

Arbuscular mycorrhizal (AM) symbiosis is one of the most prominent and beneficial plant–microbe interactions that facilitates mineral nutrition and confers tolerance to biotic and abiotic stresses. AM fungi colonize the root cortex and develop specialized structures called arbuscules where the nutrient exchange takes place. Arbuscule development is a highly controlled and coordinated process requiring the involvement of many plant proteins recruited at that interface. In contrast, much less is known about the fungal proteins involved in this process. Here, we have identified an AM fungal effector that participates in this developmental step of the symbiosis. RiCRN1 is a crinkler (CRN) effector that belongs to a subfamily of secreted CRN proteins from *R. irregularis*. CRNs have been so far only functionally characterized in pathogenic microbes and shown to participate in processes controlling plant cell death and immunity. *RiCRN1* accumulates during symbiosis establishment parallel to *MtPT4*, the gene coding for an arbuscule-specific phosphate transporter. Expression in *Nicotiana benthamiana* leaves and in *Medicago truncatula* roots suggest that RiCRN1 is not involved in cell death processes. RiCRN1 dimerizes and localizes to nuclear bodies, suggesting that, similar to other CRNs, it functions in the plant nucleus. Downregulation of *RiCRN1* using host-induced gene silencing led to an impairment of the symbiosis in *M. truncatula* and to a reduction of *MtPT4*, while ectopic expression of *RiCRN1*, surprisingly, led to a drastic reduction in arbuscule size that correlated with a decrease not only in *MtPT4* but also in *MtBCP1*, a marker for initial stages of arbuscule development. Altogether, our results suggest that a tightly regulated expression in time and space of *RiCRN1* is critical for symbiosis progression and for the proper initiation of arbuscule development.

## Introduction

Most microbial activity in soils is concentrated in the rhizosphere, the area in close vicinity to the root and influenced by the secretion of plant substances. The microbial activity of the rhizosphere and the balance between mutualistic and antagonistic microbial interactions ultimately determines plant health (Berendsen et al., 2012). Several rhizospheric microbes associate intimately with plant roots establishing long lasting relationships. Among those, the mutualistic arbuscular mycorrhizal (AM) symbiosis is the most prominent microbial association of roots involving the majority of terrestrial plants (Smith and Smith, 2011). AM fungi belong to the subphylum Glomeromycotina, from the Mucoromycota phylum (Spatafora et al., 2016) and comprise many different genera and species all forming the same type of symbiotic association. AM fungal colonization is restricted to the epidermis and the cortex of the root but excluding the endodermis. After penetration of the epidermis, the AM fungus grows into the root cortex, inter- and intracellularly, but never invading the plant protoplast. In inner cells of the cortex, intracellular tree-like branched hyphae are formed called arbuscules that are key for the nutrient exchange with the plant (Luginbuehl and Oldroyd, 2017; MacLean et al., 2017). The establishment and functioning of the AM symbiosis is accompanied by an extensive and complex bidirectional signal exchange that redirects plant and fungal development (Lanfranco et al., 2018). Interestingly, signal exchange between AM fungi and their host plants starts prior physical contact as it has been shown by transcriptomic changes in host plants and in the fungi in response to diffusible signals (MacLean et al., 2017; Lanfranco et al., 2018), and likely continues throughout the whole duration of the symbiosis. The identification of fungal signals involved in AM symbiosis has been an active field of research in the last decade with the identification of chito- and lipochito-oligosaccharides as key molecules involved in this interkingdom communication (Maillet et al., 2011; Genre et al., 2013). Perception of these signals, and likely others not yet identified, leads to the activation of the common symbiosis signaling pathway (SYM pathway) that controls the accommodation of the fungus within the cortex (Luginbuehl and Oldroyd, 2017; MacLean et al., 2017). A totally different class of fungal signal molecules has been recently identified. AM fungi, similar to pathogenic microbes have been shown to contain effector molecules in their genetic repertoire (Kloppholz et al., 2011; Tisserant et al., 2013; Lin et al., 2014; Sedzielewska Toro and Brachmann, 2016; Kamel et al., 2017; Zeng et al., 2018). Although only few functional analyses have been carried out with AM fungal effectors (Kloppholz et al., 2011; Tsuzuki et al., 2016), it is proposed that these effector proteins might contribute to modulate the immune system of the plant and/or to facilitate the nutrient exchange as it is the case in plant-pathogenic fungi (Doehlemann et al., 2014). Thus, the *R. irregularis* SP7 (Secreted Protein 7) was shown to positively impact on the symbiosis, counteracting the function of the pathogenesis-related transcription factor MtERF19 (Kloppholz et al., 2011). Also, strigolactone induced secreted protein 1 (SIS1), another effector from *R. irregularis* was shown to be induced during symbiosis and upon strigolactone treatment. Its silencing via HIGS led to a suppression of colonization and formation of stunted arbuscules (Tsuzuki et al., 2016). But, it is also conceivable that some AM fungal effectors might contribute to the plant developmental changes required for symbiosis establishment.

Among the identified AM fungal effectors in the work of Lin et al. (2014), 42 sequences were identified with similarities to proteins belonging to the large pathogen associated crinkler (CRN) effector family, specifically to the highly conserved amino-terminal (N-terminal) LFLAK domain. Among these, some also harbor a signal peptide for secretion and other conserved CRN domains. CRNs were first described in phytopathogenic oomycetes such as *Phytophthora* spp., as a large family of effectors located, together with the RxLR effectors, in fast-evolving genomic regions (Torto et al., 2003; Haas et al., 2009; Raffaele et al., 2010; Schornack et al., 2010; Amaro et al., 2017). This gene family is also present in other oomycetes such as the legume pathogen *Aphanomyces euteiches* that, interestingly, lacks RxLR effectors in its genome (Gaulin et al., 2008, 2018; Amaro et al., 2017). CRNs are in fact, ubiquitously present in all plant pathogenic oomycetes analyzed (Adhikari et al., 2013; Shen et al., 2013), but surprisingly absent in the genome of animal pathogenic ones. It has been suggested that this is an indication of their involvement in facilitating plant susceptibility, as new transcriptomic data seems to indicate (Gaulin et al., 2018). Interestingly, CRNs are also found outside the oomycetes, in several fungi including the animal pathogenic chytrid *Batrachochytrium dendrobatidis* (Raffaele et al., 2010; Sun et al., 2011) as well as in other chytridiomycota (Farrer et al., 2017). Furthermore, recent data suggests that they might be more ubiquitously distributed than predicted, and present even in non-parasitic free-living eukaryotes including plants (Zhang et al., 2016).

Like RxLR effectors, CRN effectors are modular proteins. The N-terminus harbors the characteristic LFLAK domain, with the highly conserved LxLFLAK motif and a neighboring DWL domain, marking the end of the N-terminus with the highly conserved HVLxxP motif. The carboxy-terminal (C-terminal) region exhibits a large variety of domains and it has been suggested, that recombination between different clades drives CRN diversity (Haas et al., 2009). Extensive studies on the functional role of CRN proteins have been carried out in plant pathogenic oomycetes, where they have been described as effector proteins that enter the plant cell nucleus to exert their function (Schornack et al., 2010; van Damme et al., 2012; Stam et al., 2013b; Rajput et al., 2014, 2015; Mafurah et al., 2015; Song et al., 2015). Although initially CRN proteins were characterized as cell death inducing factors (Torto et al., 2003), many studies on this subject have proven even the opposite function (Liu et al., 2011; Shen et al., 2013; Rajput et al., 2014, 2015). Thus, for instance, PsCRN115 is able to suppress the cell death elicited by PsCRN63, although these two effectors are only different in four amino acids (Liu et al., 2011). Furthermore, it was shown that those two CRN proteins interact with plant catalases to regulate plant programmed cell death by modulating H_2_O_2_ homeostasis and thus overcoming host immunity (Zhang et al., 2015). Another CRN protein shown to suppress cell death is PsCRN161. This effector, in addition, enhances tolerance to salinity and drought stress, extending the role of CRNs to the protection of plants from biotic and abiotic stresses (Rajput et al., 2015). A very recent study has even shown that the virulence activity of the *Phytophthora capsici* CRN83_152 *in planta* is not related to its cell death inducing activity (Amaro et al., 2018). All these results suggest that CRN proteins could have other functions besides cell death induction.

While it has been shown that CRN translocation into plant cells is mediated by the LxLFLAK motif in the N-terminus (Schornack et al., 2010), the effector function of CRN proteins within the plant is ascribed to their C-terminal region (Liu et al., 2011; van Damme et al., 2012; Stam et al., 2013b). Very few CRNs were predicted to contain domains with a putative function and for some of them this function has been proven. Thus, for instance, PiCRN8 C-terminus has homology to serine/threonine kinases and it was demonstrated to have an autophosphorylation activity essential for *P. infestans* virulence (van Damme et al., 2012). Similarly, the PsCRN108 from *P. sojae* contains a Helix hairpin Helix (HhH) motif suggesting that CRNs could modulate plant gene expression by targeting plant promoters. Indeed, in PsCRN108 this domain was responsible for inhibition of heat shock proteins expression by binding to their promoter and preventing the association of the corresponding heat shock transcription factor (Song et al., 2015). Also, the HhH-like endonuclease motif in AeCRN13 from the legume pathogen *A. euteiches* was shown to be essential for DNA binding *in planta*. CRN13 binding to DNA induces the DNA damage response eventually leading to cell death (Ramirez-Garces et al., 2016). Interestingly AeCRN13 is homolog to BdCRN13 from the animal pathogenic fungus *B. dendrobatidis*, and reciprocal expression in amphibians or plant cells induced aberrant cell development, suggesting a conserved mechanism of function for both effectors.

Haas et al. (2009) proposed a CRN classification in families grouped by the homology of their C-termini and new domains were proposed. However, no functions could be ascribed to those domains. In contrast, the new non-targeted analysis by Zhang et al. (2016) that extended the presence of CRNs to non-pathogenic organisms, allowed a re-classification of CRN proteins and a plethora of new functional domains were identified in their C-termini. These results have challenged our view of CRN proteins and will allow to propose new hypotheses regarding their evolution and function (Amaro et al., 2017).

In this work, we carried out the first characterization of a CRN protein from the AM fungus *R. irregularis*. We show that a subset of *R. irregularis* CRNs with a predicted signal peptide is expressed during plant colonization. From those, *RiCRN1* accumulates concomitantly to the arbuscule marker *MtPT4*. Localization of RiCRN1 was observed in nuclear bodies and silencing of the gene led to an impairment of fungal colonization. Most interestingly, ectopic expression of *RiCRN1* in *Medicago truncatula* showed a drastic arbuscule phenotype. Our data suggest a critical involvement of RiCRN1 proteins in the establishment of a functional symbiosis.

## Materials and Methods

### Plant Material and Growth Conditions

*Medicago truncatula* Jemalong A17 plants were grown at 25°C in a growth chamber (Binder GmbH, Germany) with a 16-h light/8-h darkness period. To generate composite plants, *M. truncatula* roots were transformed with *Agrobacterium rhizogenes* ARquaI (Quandt et al., 1993) after the protocol of Boisson-Dernier et al. (2001) and cultivated on Fahraeus medium for 5 weeks. Successfully transformed roots were visually selected via constitutive expression of DsRED. Wild type roots were removed, and composite plants were cultivated one additional week on Fahraeus medium containing 400 mg/l Augmentin (AmoxiClav, Hikma Farmaceutical, Portugal). Plants were then transferred into 50 ml Falcon tubes containing a sand:gravel (1:4) mixture inoculated with *R. irregularis* DAOM 197198 that was previously propagated in monoaxenic culture with carrot roots (1 plate inoculum for 100 ml substrate). Plants were cultivated for 5 weeks and fertilized with a half strength low Pi (20 μM) Long Ashton nutrient solution (Hewitt, 1966) twice a week (5 ml). Wild type *M. truncatula* plants used for time course analyses were inoculated and grown under the same conditions.

*Nicotiana benthamiana* was used for transient expression of GFP fusion proteins in localization and necrosis analyses. Plants were grown in soil at 28°C in a growth chamber (CLF Plant Climatics, Germany), with a 16-h light/8-h darkness period.

*Rhizophagus irregularis* DAOM 197198 (Schenck and Smith, 1982) was cultivated in monaxenic culture as described before (Kuhn et al., 2010).

### Bioinformatic Online Resources Used in This Study

Signal peptide prediction analyses for RiCRN protein candidates were carried out using SignalP3.0^1^ and SignalP4.0^2^ versions. All sequences that were predicted to contain a signal peptide in at least one of the servers were considered as putative secreted effectors. The presence of possible NLS in RiCRN candidates was predicted using PSORTII^3^. Alignments were performed with the ORF (full length, N-terminal, or C-terminal regions) of all RiCRNs using Clustal Omega^4^ and visualized with Jalview^5^. N-terminal regions were defined as the amino acid sequence located in front of the characteristic HVLxxP motif as previously described in Haas et al. (2009). For analyses of consensus motifs, the respective aligned (ClustalO) sequences were used in WebLogo^6^ to create consensus logos. For protein structure homology-modeling of RiCRN1 and PcCRN20, the Phyre2 web portal^7^ was used implementing the intensive modeling mode option (Kelley et al., 2015). The structural models obtained were visualized and manually curated in UCSF Chimera by removing the low confident part of the proteins. Superimposed models were created using the integrated MatchMaker tool (Pettersen et al., 2004). Template structures used by Phyre2 were obtained directly from PDB^8^.

The multiple sequence alignment of RiCRN1 and RiCRN10 with the CR-REase 5 family was manually performed using the same multiple sequence alignment created by Zhang et al. (2016).

### Generation of Constructs Used in This Study

For overexpression analyses in *M. truncatula* roots, the carboxy terminus of *RiCRN1* was amplified from cDNA (starting right after the HVLVEPP motif), subcloned into pENTR^®^/D-TOPO (Invitrogen by Thermo Fisher Scientific, Germany) and cloned into the destination vector 2xP35S-pKGW-RedRoot (Heck et al., 2016). For yeast two hybrid interaction studies, the ORF of *RiCRN2* (without signal peptide), *MtSR45*, *MtU1-70k*, and *RiCRN1* (without signal peptide) as well as the *RiCRN1* carboxy terminus were amplified from cDNA using restriction sites, subcloned into PCR^®^2.1 TOPO^®^ (Invitrogen by Thermo Fisher Scientific, Germany) and cloned into the bait or prey vectors pGBKT7 or pGADT7 (Takara Clontech Bio Europe, France). *MtU2AF^35b^* was isolated from a previous yeast two hybrid library screen (Kloppholz et al., 2011). For localization and necrosis analyses in *N. benthamiana*, the ORF of *RiCRN1*, with and without signal peptide, as well as the C-terminus alone, were amplified from cDNA and subcloned into pENTR^®^/D-TOPO (Invitrogen by Thermo Fisher Scientific, Germany). For localization studies, the constructs were cloned into the destination vector pCGFP-RR (Kuhn et al., 2010) and for necrosis assays, constructs were cloned into pK7FWG2 (Karimi et al., 2002). For co-localization studies, the carboxy terminus of *RiCRN1* was cloned into the vector pK7FWG2 (Karimi et al., 2002) and *MtSR45* was cloned into the vector pK7RWG2 (Limpens et al., 2005). Silencing of *RiCRN1* was carried out by host-induced gene silencing (HIGS) as described in Helber et al. (2011). Three RNAi constructs targeting the regions from -119 to +200 (RiCRN1.1), +52 to +353 (RiCRN1.2), and +209 to +514 (RiCRN1.3) with respect to the start codon ATG were amplified from cDNA, subcloned into pENTR^®^/D-TOPO (Invitrogen by Thermo Fisher Scientific, Germany) and then cloned into pK7GWIWG2D (Karimi et al., 2002). All primers used for cloning of the constructs described above, are listed in **Supplementary Table S2** and the three target sequences of *RiCRN1* HIGS constructs are listed in **Supplementary Table S3**.

### Gene Expression Analyses

Total RNA extraction from *M. truncatula* roots was performed using the innuPREP RNA kit (Analytik Jena AG, Germany) and quantified using a DeNovix Ds-11+Spectrophotometer (DeNovix Inc., United States). cDNA synthesis was performed using the reverse transcriptase SuperScriptII (Invitrogen by Thermo Fisher Scientific, Germany) as described before (Kuhn et al., 2010). Quantitative real-time expression analyses were carried out using the MESA Green 231qPCR Master Mix Plus (Eurogentec, Germany) in a CFX Connect Real-Time PCR Detection System (Bio-Rad Laboratories GmbH, Germany). A total of 1 μl cDNA (1:5) was used per well as template with the following PCR protocol: 5 min 95°C, 15 s 95°C, 20 s 56°C, 30 s 72°C (40 cycles). Plant transcript levels and transcript levels of the translation elongation factor 1-alpha of *R. irregularis* (*RiTEF1α*, DQ282611) were normalized to the translation elongation factor 1-alpha of *M. truncatula* (*MtTEF1α*, Medtr6g021800) while fungal transcripts were normalized to *RiTEF1α*. Transcript levels of genes were determined in three technical replicates in each independent biological replicate. Numbers of biological replicates are indicated in the corresponding figure legends. All primers are listed in **Supplementary Table S2**.

### Yeast Two Hybrid Interaction Studies

Direct protein–protein interactions were assayed by co-transforming the bait (pGBKT7) and prey (pGADT7) vectors in *Saccharomyces cerevisiae* AH109 (Takara Clontech Bio Europe, France). To verify successful transformation events, single colonies were plated in a dilution series, on yeast synthetic drop-out (SD) medium, lacking leucine, and tryptophan. Interaction was tested on SD medium deficient of leucine, tryptophan, histidine, and adenine. Yeast growth was validated after 5 days at 30°C.

### Transient Expression of Proteins in *N. benthamiana*

For localization and necrosis assays *N. benthamiana* leaves were infiltrated with *A. tumefaciens* GV3101 or AGL1 strain, respectively, after the protocol of Voinnet et al. (2003). The bacterial suspension was infiltrated in leaves using a needleless syringe. For localization studies, the bacterial suspension was infiltrated into the whole leaf area, with a final optical density (OD_600_) of 0.5 (adjusted with AS medium supplemented with 2% sucrose). Plants were cultivated at 21°C and confocal microscopy was performed 3 days after infiltration. For the necrosis assay, bacterial culture was infiltrated only in spots into the leaves, using an OD_600_ of 0.25 (adjusted with AS medium without sucrose). Plants were cultivated at 21°C and scoring of lesions was performed 7 days after infiltration. To verify protein integrity in *N. benthamiana* during necrosis analyses, a western blot was employed to detect RiCRN1:eGFP (81.3 kDa), RiCRN1ΔSP:eGFP (79.7 kDa), RiCRN-C:eGFP (78 kDa), eGFP:PcCRN20_624 (58.4 kDa), and eGFP:PcCRN1_719 (81.5 kDa) or free eGFP (26.9 kDa). *N. benthamiana* leaves expressing these constructs, as well as untransformed leaves, were ground to a fine powder in liquid nitrogen. Total protein extraction was performed using a GTEN extraction buffer [25 mM Tris–HCL (pH7.5), 150 mM NaCl, 1 mM EDTA, 10% Glycerol, 1X protease inhibitor cocktail (complete^TM^, Roche), 10 mM DTT, 1 mM PMSF and 0.2% Nonidet P40]. Extracts were spun down, or filtrated (RiCRN1:eGFP) through Miracloth (Calbiochem, Germany) and supernatants/flow-through (RiCRN1:eGFP) was separated in SDS-PAGE (5% stacking-gel and 8% running-gel) and blotted on nitrocellulose membranes. Ponceau S staining (Sigma-Aldrich, Germany) was used to show transfer of the protein to the membrane. Immunodetection of GFP was carried out with an anti-GFP primary antibodies from rabbit (G1544, Sigma-Aldrich, Germany), in a 1:4000 dilution and a horseradish peroxidase coupled anti-rabbit secondary antibody from goat (A0545, Sigma-Aldrich, Germany) in a 1:8000 dilution. Chemiluminescence was detected with the ChemiSmart 5100 detector (Peqlab Biotechnology GmbH, Germany).

### Phenotypical Analysis and Quantification of Mycorrhization

Fungal structures were immunostained with WGA-fluorescein as described in Rech et al. (2013) for phenotypical analysis and quantification of mycorrhization. Quantification of mycorrhizal structures was carried out according to Trouvelot et al. (1986). F% represents the frequency of mycorrhization, M% the intensity of colonization, A% the abundance of arbuscules, and I% the abundance of hyphae in the root system. For morphometrical analyses of arbuscule length and width, images were taken randomly from colonized roots. Measurements were done using the Fiji software^9^ on *n* ≥ 200 arbuscules.

### Confocal Microscopy

Microscopical analyses were done using a Leica TCS SP5 (DM5000) confocal microscope with conventional PMT detectors and the color camera Leica DFC295 (Leica, Germany). WGA-fluorescein stained roots and eGFP in localization analyses were both excited with an argon laser at 488 nm. Emission was detected from 493 to 530 nm. In co-localization studies, mRFP was excited at 561 nm with a DPSS 561 laser and emission was detected from 566 to 670 nm. Images were processed using the Fiji software^9^.

### Statistical Analyses

All data shown represent the mean of several biological replicates indicated in each figure. Error bars represent SEM. Two-tailed Student’s *t*-test was used for pairwise comparisons of qPCR data, quantified data of mycorrhizal colonization and comparison of morphometrical analysis of arbuscules. Significance is indicated by asterisks (^∗^*p* < 0.05; ^∗∗^*p* < 0.01; ^∗∗∗^*p* < 0.001). A one-way ANOVA with *post hoc* Tukey HSD Test was used to validate significance of expression between time points of the time-course. Different letters indicate significant difference in expression (*p* < 0.05). Values of *n* are indicated in the corresponding figure legends.

### Data Availability

The CDS sequences of *RiCRN1* to *RiCRN8* and *RiCRN10* have been submitted to GenBank with the identification numbers: MH542411 to MH542419.

## Results and Discussion

### *R. irregularis* Contains a Large Number of CRN-Like Proteins

Sequencing of the *R. irregularis* genome (Tisserant et al., 2013; Lin et al., 2014) showed the existence of several entries with similarities to genes coding for CRN proteins, that had been first identified in oomycetes and chytridiomycota (Haas et al., 2009; Raffaele et al., 2010). Surprisingly, the CRN sequences from *R. irregularis* identified in two different sequencing projects revealed major differences in number and in composition, indicating that perhaps the number of CRN-like sequences might be larger than the current assemblies suggest. Indeed, the analysis of two *R. irregularis* isolates (DAOM 197198 and C2) and of *G. diaphanus* (data not shown) showed the existence of many other putative CRN-like proteins lacking the LFLAK domain. Therefore, they were not ascribed as CRNs but the presence of several of the CRN C-terminal domains identified in Haas et al. (2009), such as DC or DN17, indicates that they might be *bona fide* CRN proteins. We decided therefore, to look at the different data sets publicly available (Tisserant et al., 2013; Lin et al., 2014; Chen et al., 2018) and make a global analysis of CRNs in the symbiotic AM fungus *R. irregularis*. The data sets used contained 82 and 42 sequences. Several of the genes are present in both but often not as complete sequences. Multiple ClustalO alignments showed the existence of clusters that corresponded to genes having similar amino- or carboxy-terminal domains (**Supplementary Figure 1** and **Supplementary Table S1**).

**FIGURE 1:**
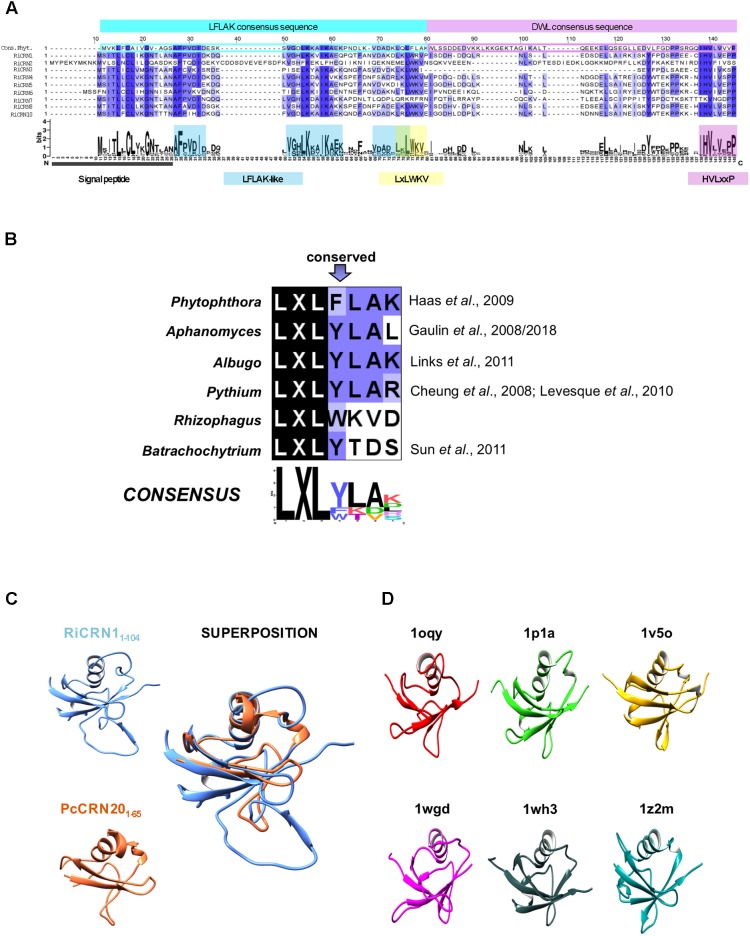
Candidate CRN effectors from *R. irregularis* containing a predicted signal peptide. **(A)** Alignment of the N-terminal regions of RiCRN proteins containing a predicted SP (SignalP-3.0 or 4.0) with the consensus sequence of the *P. infestans* LFLAK and DWL domains (Haas et al., 2009). At the position of the *P. infestans* highly conserved LxLFLAK motif, a modified version (LxLWKV) is present in almost all *R. irregularis* sequences. Moreover, most RiCRNs show two additional conserved motifs (AFPVDI and VGLKxxIKA) within the *Phytophthora* LFLAK domain. Except for RiCRN7, all *R. irregularis* members harbor the characteristic HVLxxP motif, which marks the end of *Phytophthora* N-terminal CRN region. **(B)** Comparison of LxLFLAK-like motifs present in different organisms. This motif is highly conserved in oomycetes but less conserved in fungi. The LxL triade is conserved in all organisms. **(C)** Partial three-dimensional models of the N-terminal regions of RiCRN1 and PcCRN20_624 obtained from Phyre2 show the fold of the Ubiquitin-like superfamily and can be superimposed. Amino acids included in each model are indicated. **(D)** Protein structures from the 6 PDB templates used by Phyre2 to model the N-terminus of RiCRN1 are shown. The structures belong to the ubiquitin-like domains from the human ubiquitin-like DNA repair proteins hHR23a (1ogy) and hHR23B (1p1a), the mouse hypothetical 1700011N24Rik protein (1v5o), the human Herp protein (1wgd), the human 2′-5′-oligoadenylate synthetase-like (p59 OASL) protein (1wh3) and the human Interferon-Induced ubiquitin cross reactive protein ISG15 (1z2m).

Interestingly, although very few of the CRN-like proteins from *R. irregularis* contained a signal peptide, most of them did not, and were accordingly not predicted as secreted proteins. This suggests that these proteins might exert their function within the fungal cytoplasm, or that they might be alternatively secreted. In agreement with this, in oomycetes only very few CRN proteins contain a signal peptide, and from the rest, several were predicted to contain other motifs that might serve for alternative secretion (Haas et al., 2009; Gaulin et al., 2018). We decided to first focus our attention to those *R. irregularis* CRN proteins having a predicted signal peptide and thus, more likely functioning as effectors outside the fungus. From all data sets analyzed, only nine CRN proteins were predicted as secreted according to SignalP 3.0 or SignalP 4.0. An alignment of these nine proteins using ClustalO clearly showed a highly conserved N-terminus and a more divergent C-terminus (**Supplementary Figure 2**). All amino termini contained, similar to the CRNs described in oomycetes, the LFLAK and the DWL domains, proposed as trafficking sequences for entering the plant cell (Schornack et al., 2010). Compared to the consensus N-terminus of *Phytophthora* spp. described in Haas et al. (2009), we could observe that in *R. irregularis*, the highly conserved LxLFLAK motif is modified to LxLWKV not only in the nine secreted CRN proteins but also in many other CRN sequences from both genome assemblies (**Figure 1A** and **Supplementary Table S1**). This is interesting, because deviations of the LxLFLAK motif have been also found in some oomycetes such as *Phythium* or *Aphanomyces*, and even more dissimilar in the chytridiomycete *B. dendrobatidis* (Cheung et al., 2008; Gaulin et al., 2008, 2018; Levesque et al., 2010; Links et al., 2011). The consensus logo of all these organisms for the LxLFLAK motif shows that only the LxL triade is fully conserved (**Figure 1B**). Interestingly, besides the LxLWKV motif, *R. irregularis* CRNs with signal peptide also contain two other amino acids stretches highly conserved in the LFLAK domain, and also present in the *Phytophthora* consensus sequence (AFPVxI and LKxxI). This is in marked contrast to the DWL domain, in which the conservation is mainly restricted to the conserved HVLxxP motif that in oomycetes has been defined as the amino acid stretch marking the end of the N-terminus (Haas et al., 2009). Given these commonalities and disparities, we wondered how the protein structure of RiCRN1, as a representative of the secreted AM CRNs, would correspond to PcCRN20, a CRN protein from *P. capsici*, known to elicit cell death in leaf tissue (Stam et al., 2013b). To assess this, three-dimensional models of RiCRN1 and PcCRN20 N-termini were created using the structure prediction server Phyre2 according to proteins of the β-GRASP (ubiquitin-like) domain superfamily (IPR012675) (**Figures 1C,D**). This is consistent with the predictions made by Zhang et al. (2016) that considered the N-termini of CRN proteins as header domains containing the ubiquitin-like fold. N-termini of RiCRN1 (amino acids 1–104) and PcCRN20 (amino acids 1–65) show high similarities in protein structure, with the exception of one extra loop in RiCRN1 located at the transition from the LFLAK to the DWL domain (**Figure 1C**).

**FIGURE 2:**
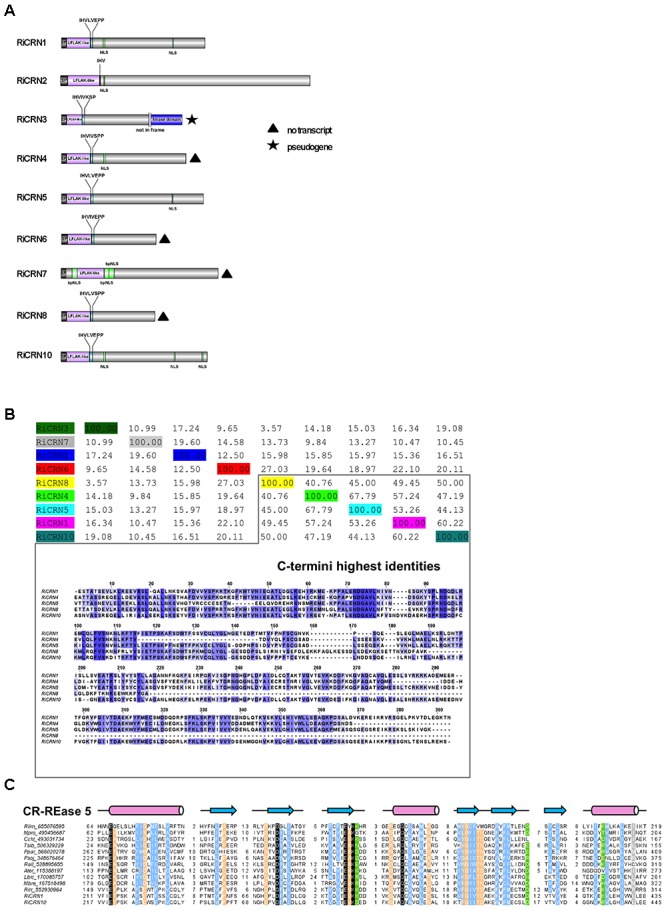
Sequence features of *R. irregularis* CRNs with predicted signal peptide. **(A)** Protein organization of secreted *R. irregularis* CRNs. Besides the N-terminus that contains the signal peptide, the LFLAK-like and DWL domain, RiCRN proteins contain C-termini of different lengths, some of them harboring one or more predicted NLS motifs (PSORTII), sometimes as bipartite NLSs (bpNLS). Four RiCRN candidates (*RiCRN4*, *6*, *7*, and *8*) did not show expression during symbiosis and are marked with a black triangle (see **Supplementary Figure 3**). RiCRN3 is predicted to be a pseudogene because its transcript contains a premature stop codon that would prevent the translation of the kinase domain located at the end of the C-terminus. **(B)** Percentage identity matrix score (ClustalO) of the secreted RiCRN C-termini. Five of them show high protein identities (between 41 and 68%), while the others are less conserved. Alignment of the C-termini (ClustalO, identical amino acids are shown in blue) revealed highly conserved amino acid stretches, suggesting that they might belong to a common CRN subfamily. **(C)** Multiple sequence alignment of the CR-REase 5 family members used in Zhang et al. (2016) together with the RiCRN1 and RiCRN10 showed the presence of the conserved catalytic residues (in black) and the topology predicted for the CR-REase 5 family. Pink cylinders indicate α-helices and blue arrows indicate β-sheets. Sequence limits and missing residues between sequence blocks shown are indicated with numbers.

The analysis of the C-termini of secreted *R. irregularis* CRN proteins showed that 6 of 9 contained at least one nuclear localization domain (NLS), in some cases more, like RiCRN10 that contained three (**Figure 2A**). This is similar to the presence of NLS in about 60% of oomycetes CRNs, and consistent with the nuclear localization observed for most CRN proteins analyzed (Haas et al., 2009; Schornack et al., 2009; Gaulin et al., 2018). Besides the NLS, three *R. irregularis* CRNs (RiCRN4, RiCRN5, and RiCRN10) were previously predicted to contain the DN17 domain (Lin et al., 2014) while RiCRN2 was shown to contain the DC domain (Tisserant et al., 2013; Lin et al., 2014; Chen et al., 2018). However, none of these two domains have a clearly ascribed function. In contrast, most of the secreted *R. irregularis* CRNs contained functional domains such as REases (Restriction Endonucleases 5, 6, and 8), NTPase4 and HhH domains (**Supplementary Table S1**) according to the prediction by Zhang et al. (2016).

Using a percentage of identity matrix for all predicted secreted *R. irregularis* CRNs, it could be observed that five of them (RiCRN1, RiCRN4, RiCRN5, RiCRN8, and RiCRN10) showed a high conservation in their C-terminus (**Figure 2B**). From those, three of them (RiCRN5, RiCRN8, and RiCRN10) were annotated as containing an REase5 domain, while RiCRN4 was, in contrast, predicted to contain an REase6 domain due to the presence of an extra amino acid stretch (Zhang et al., 2016). RiCRN8 is a shorter sequence than the rest and despite the high homology to RiCRN5 and RiCRN10, its potential REase5 domain is likely not detectable. RiCRN1 was not included in the analysis of Zhang et al. (2016). Therefore, in order to investigate whether RiCRN1 could also contain an REase5 domain, we aligned it with the sequences from the REase5 family used in the Zhang analysis. The results showed, that indeed, all critical amino acids, including those of the catalytic domain, are conserved in RiCRN1. Therefore, we hypothesize that this protein also contains an REase5 domain (**Figure 2C**).

### CRN1 a Novel Effector Protein From *R. irregularis*

In order to investigate the role that predicted secreted CRN-like proteins might play in *R. irregularis* during the interaction with plants, we first analyzed their expression during symbiosis. From those nine CRN genes, several were not expressed at all or only at low levels in *M. truncatula* mycorrhizal roots (RiCRN3, RiCRN4, RiCRN6, RiCRN7, and RiCRN8). In contrast, RiCRN1, CRN2, CRN5, and CRN10 showed relatively high levels of expression *in planta* (**Supplementary Figure 3**). We could also detect a possible CRN-like pseudogene in this group, RiCRN3, that contains a premature stop codon and a predicted kinase domain not in frame, suggesting that some CRN-like proteins might be subjected to rapid evolution as it has been shown for *Phytophthora* (Haas et al., 2009).

**FIGURE 3:**
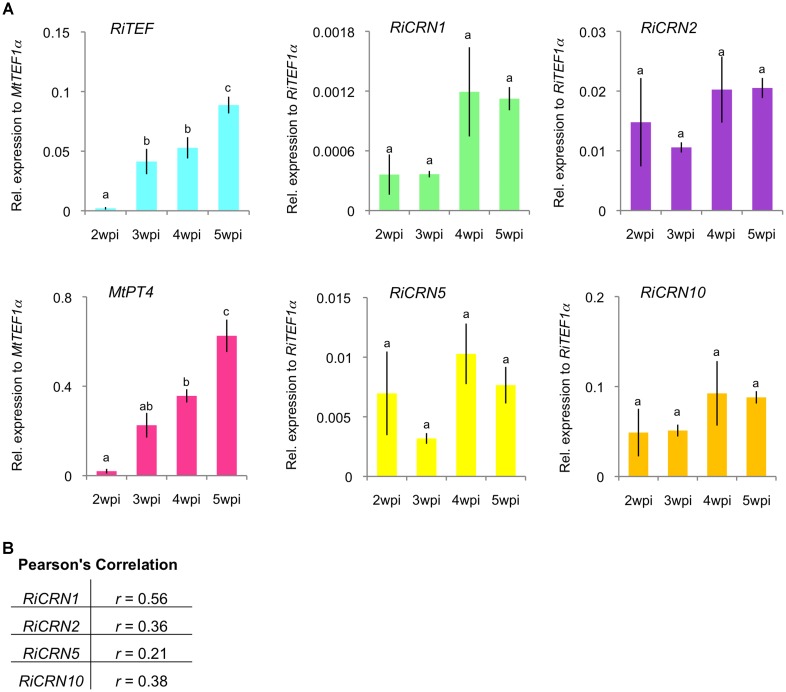
Expression analysis of a subset of *R. irregularis* CRN effectors during symbiosis with *M. truncatula*. A time course experiment was carried out to analyze the expression of *RiCRN1, RiCRN2*, *RiCRN5*, and *RiCRN10* during symbiosis of *R. irregularis* with *M. truncatula*. **(A)** Expression of the *R. irregularis* elongation factor 1α (*RiTEF1α*), the phosphate transporter and arbuscule marker *MtPT4*, and the *R. irregularis* CRN effectors *CRN1*, *CRN2*, *CRN5*, and *CRN10* were analyzed at 2, 3, 4, and 5 weeks post inoculation (wpi). Expression is shown as relative expression to *M. truncatula* elongation factor 1α (*MtTEF1α*) for *MtPT4* and *RiTEF1α* or to *RiTEF1α* for *RiCRN1*, *RiCRN2*, *RiCRN5*, and *RiCRN10*. All four CRNs are expressed during the symbiosis, albeit with different patterns. Error bars represent SEM with *n* = 3 biological replicates for all time points. A one-way ANOVA with *post hoc* Tukey HSD test was used to validate significance of expression between time points. Different letters indicate significant difference in expression (*p* < 0.05). **(B)** A Pearson’s correlation analysis was carried out between expression of *MtPT4* and the *R. irregularis CRN1*, *CRN2*, *CRN5*, and *CRN10. RiCRN1* is the only CRN gene showing a moderate correlation (*r* ≥ 0.50) with the arbuscule marker *MtPT4.*

The expression of *RiCRN1*, *2*, *5*, and *10* was further analyzed in a time-course manner in *M. truncatula* plants inoculated with *R. irregularis*. Plants were harvested at 2, 3, 4, and 5 weeks post inoculation (wpi) and the fungal colonization levels assessed by the expression of the fungal marker *RiTEF1α*. The level of functional symbiosis was determined by the expression of the phosphate transporter *MtPT4* which is a key marker for arbuscule-containing cells and its function directly relates to the symbiotic phosphate download that takes place at these structures (Harrison et al., 2002; Javot et al., 2007). The four CRN-like genes analyzed showed different patterns of expression with *RiCRN1* accumulating progressively during symbiosis (**Figure 3A**). A Pearson’s correlation analysis with *MtPT4* showed that only *RiCRN1* had a moderate positive correlation to this marker, while it is difficult to predict a trend for the other three genes (**Figure 3B**). This is interesting because CRNs from *Phytophthora* have been shown to have differential patterns of expression during infection. Thus, for instance CRNs from *P. capsici* can be divided in two groups according to their expression patterns, with Class 1 upregulated in early and late stages of colonization and Class 2 with expression that gradually increases to peak in late stages (Stam et al., 2013a,b). In *Phytophthora*, a correlation with the presence of the DN17 C-terminal domain and an effector expression at later stages was observed (Stam et al., 2013a,b). However, RiCRN5 and RiCRN10, which presumably contain the DN17 domain and are highly similar to RiCRN1, do not show this expression trend. Thus, it seems unlikely that the presence of the DN17 is related to the timing of expression in AM fungi. CRN effectors have been so far only functionally analyzed in pathogenic organisms, therefore, and given its pattern of expression, we decided to investigate the function of RiCRN1 during the mycorrhizal symbiosis.

### RiCRN1 Localizes to Nuclear Bodies

**FIGURE 4:**
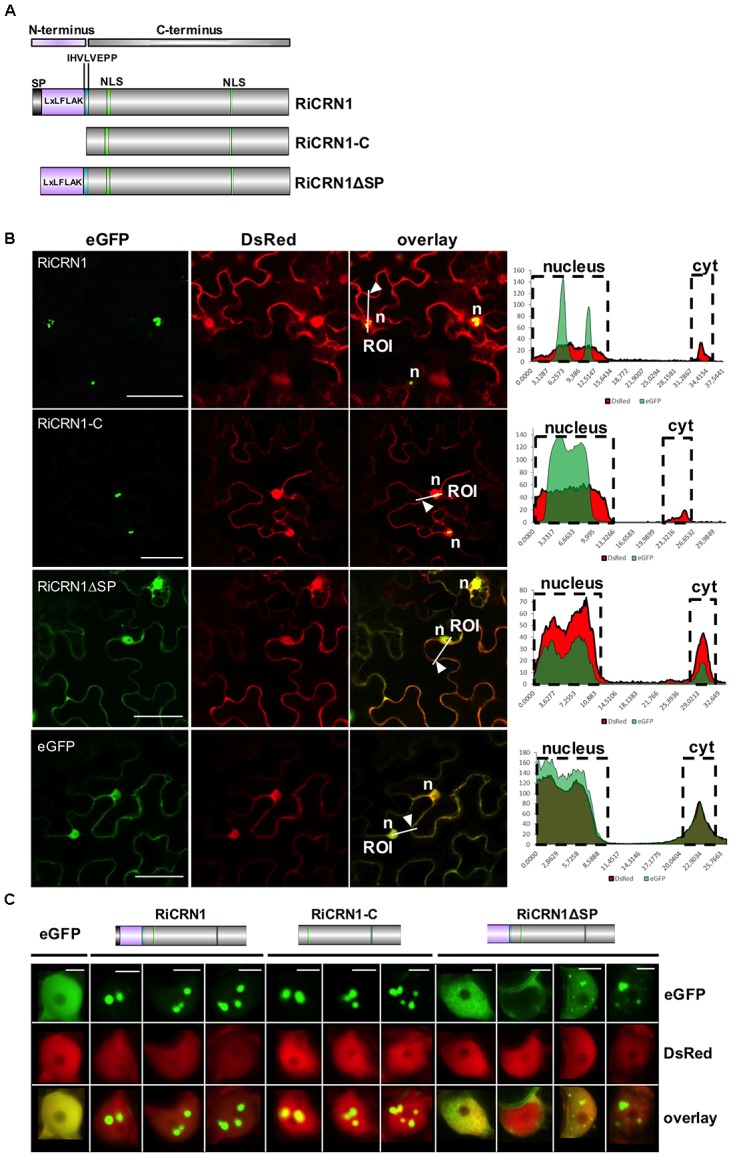
RiCRN1 localizes to the plant nucleus. **(A)** Domain organization of RiCRN1 constructs employed for localization assays. **(B)** Confocal imaging shows localization of ectopically expressed RiCRN1 versions fused to eGFP in *N. benthamiana* epidermal cells. Free DsRed was co-expressed as control for transformation and labels the plant nucleus and cytoplasm. RiCRN1:eGFP and RiCRN1-C:eGFP localize to distinct nuclear foci. No or only a weak signal is visible in the cytoplasm. In contrast, RiCRN1ΔSP:eGFP localizes to the cytoplasm and nucleus similar to free eGFP. Right panels show fluorescence intensity at specific transects marked in the overlay pictures. ROI indicates start of transection lines for fluorescent intensity measurements. White arrowheads indicate cytoplasmic areas. *n* = nucleus. Scale bar = 50 μm. **(C)** Representative localization pictures of the three RiCRN1 versions in nuclei. RiCRN1:eGFP as well as RiCRN1-C:eGFP localize to discrete nuclear foci, while RiCRN1ΔSP:eGFP shows variable nuclear patterns: even nuclear distribution, localization in foci or absence from the nucleus. Scale bar = 5 μm.

To date, all functionally characterized CRN effector proteins have been shown to localize to the nucleus when expressed *in planta* (Haas et al., 2009; Schornack et al., 2010; Stam et al., 2013a,b; Mafurah et al., 2015; Song et al., 2015; Zhang et al., 2015). Interestingly, distinct nuclear localization patterns could be observed for different CRN proteins, suggesting that they might not all perform the same function (Stam et al., 2013a,b). In order to analyze whether RiCRN1, that possesses two predicted NLS, also localizes to the cell nucleus and if yes to which region, the protein was tagged with GFP at its carboxy-terminus. Localization analyses were performed in *N. benthamiana* leaves. Three different versions of the protein were employed, the full-length (RiCRN1:eGFP), the carboxy-terminus starting after the HVLxxP-like motif (RiCRN1-C:eGFP) and the full-length without secretion peptide (RiCRN1ΔSP:eGFP) (**Figure 4A**). Confocal microscopy analyses showed that RiCRN1 and RiCRN1-C were exclusively observed in nuclear bodies (**Figure 4B**). In contrast, expression of RiCRN1 without signal peptide but still containing the LFLAK-like domain (RiCRN1ΔSP:eGFP) showed cytoplasmic and nuclear localization, with variable patterns and only sometimes at nuclear bodies (**Figure 4C**). This suggests that the presence of the putative entry domain (LFLAK domain) might perturb protein localization. We hypothesize that RiCRN1 in the natural context localizes to nuclear bodies and that the C-terminus is responsible and sufficient for this localization. This is in line with results from oomycete CRNs that show that the C-terminus is sufficient to target the proper nuclear localization and for the induction of cell death (Schornack et al., 2010; Stam et al., 2013b). Our results also suggest that RiCRN1 is able to enter the plant cell on its own without the requirement of additional fungal proteins.

### RiCRN1 Forms a Homodimer

**FIGURE 5:**
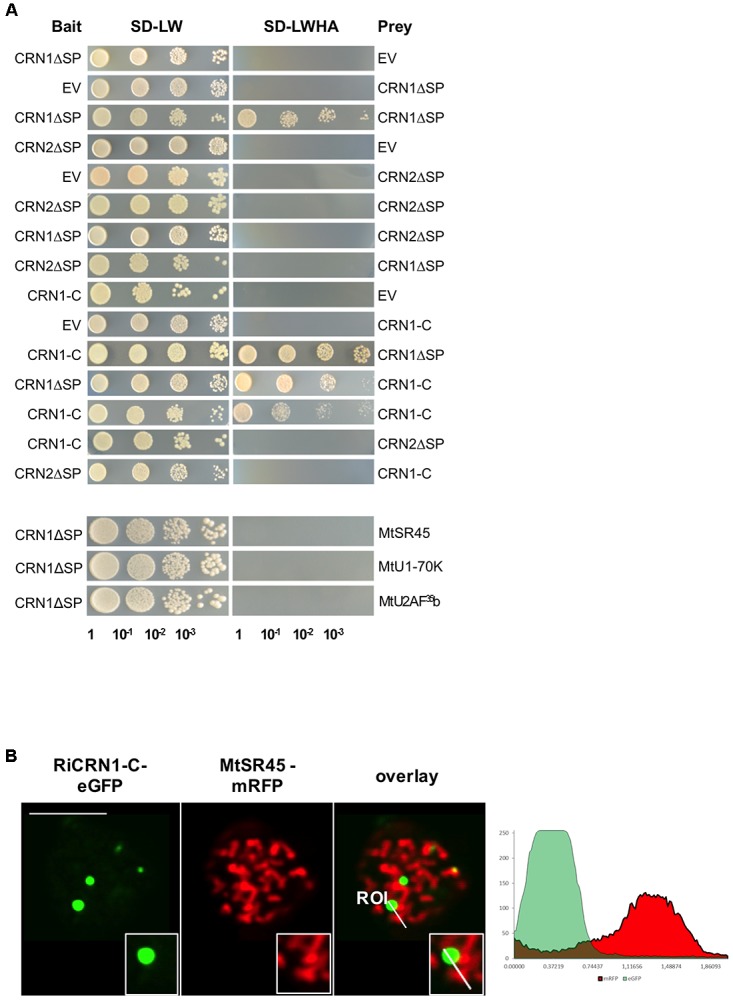
RiCRN1 forms a homodimer but does not interact neither with RiCRN2 nor with several splicing factors. **(A)** A direct interaction assay was carried out using the Y2H system. RiCRN1ΔSP was tested for interaction with itself, RiCRN2ΔSP and the splicing factors MtSR45, MtU1-70K, and MtU2AF^35^. In addition, RiCRN1-C was assayed for interaction with itself, RiCRN1ΔSP and RiCRN2ΔSP. Successful transformation was confirmed by growth on SD-LW medium in serial dilutions. Positive interaction was visualized on SD-LWHA medium. RiCRN1 is able to form a homodimer but does not interact with RiCRN2ΔSP or any of the tested splicing factors. **(B)** A co-localization experiment was carried out in *N. benthamiana* leaves with ectopically expressed RiCRN1-C fused to eGFP and the splicing factor MtSR45 fused to mRFP. Confocal imaging shows localization of both fusion proteins in distinct nuclear foci, however, strongly excluding each other. Right panel shows fluorescence intensity at specific transects marked in the overlay pictures. ROI indicates start of transection lines for fluorescent intensity measurement. Scale bar = 10 μm.

Some CRN proteins have been shown to form homo and hetero-dimers and this process to be essential for their function at controlling plant immunity (Li et al., 2016). Thus, homodimerization of PsCRN63 was shown to be critical for suppressing immunity and induction of cell death. Interestingly, PsCRN63 can also heterodimerize with PsCRN115, which is only 4 amino acids different, as well as with two other dissimilar CRN proteins. PsCRN115 has an opposed function to PsCRN63 and suppresses host defenses (Liu et al., 2011). Thus, it is possible that homo- and heterodimerization processes among CRNs could be critical to control the output of effector functions in plants (Amaro et al., 2017). In order to investigate whether RiCRN1 could form homo- or heterodimers with a different type of CRN protein, yeast two hybrid analyses with RiCRN1 and RiCRN2 were carried out (**Figure 5A**). Interestingly, RiCRN1 expressed without signal peptide, and in contrast to RiCRN2, was able to dimerize. However, both proteins did not interact with each other. Noteworthy, RiCRN1-C was sufficient to confer homodimerization, suggesting that dimerization could be important for RiCRN1 function at nuclear bodies. Nuclear bodies are often occupied by splicing components such as U1-70K, U2AF^35^b, or SR proteins and are then called speckles (Ali et al., 2008; Day et al., 2012). Therefore, in order to test whether RiCRN1 could be part of the splicing machinery, interaction assays with those splicing components were also analyzed by yeast two hybrid (**Figure 5A**). The results showed that RiCRN1 did not interact, at least directly, with any of those components. Furthermore, co-localization experiments with MtSR45 showed localization of the proteins in different nuclear bodies strongly excluding each other (**Figure 5B**). Altogether, it appears that RiCRN1 might play a role *in planta* as a dimer but functionally not related to splicing.

### RiCRN1 Does Not Control Plant Cell Death

**FIGURE 6:**
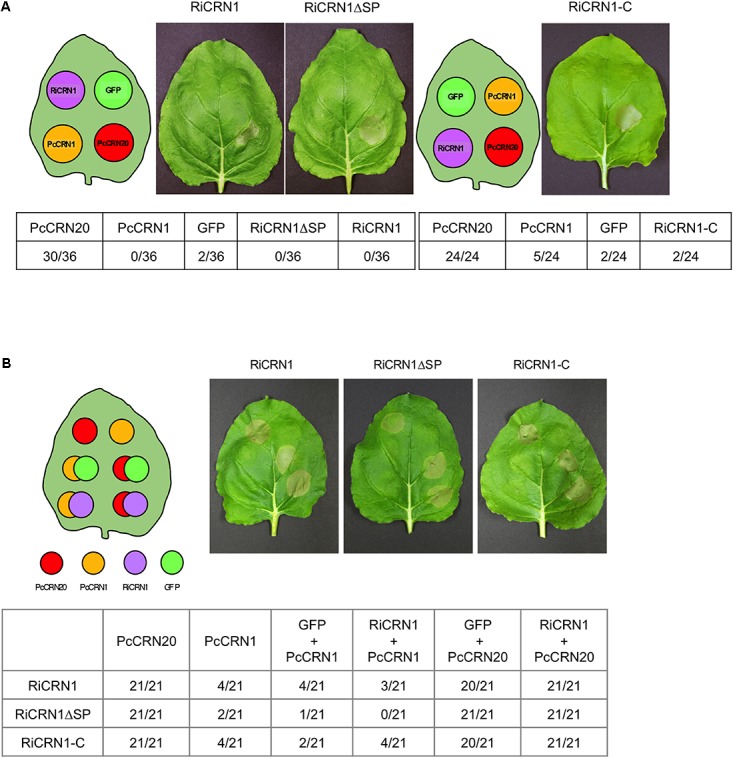
RiCRN1 does not control cell death in *N. benthamiana.* **(A)** To test a potential ability of cell death induction, a necrosis assay was carried out in *N. benthamiana*. For transient expression, leaves were infiltrated with *A. tumefaciens* carrying the constructs RiCRN1:eGFP, RiCRN1ΔSP:eGFP and RiCRN1-C:eGFP as well as PcCRN20_624 (positive control), PcCRN1_719 (negative control) and eGFP (negative control). Photographs were taken 7 days after infiltration. Quantification is shown as numbers of leaves displaying necrosis compared to the total amount of leaves assayed per construct. Infiltration sites of the three RiCRN1 versions show healthy leaf tissue with no induced cell death. **(B)** To assay a putative function of RiCRN1 as cell death inhibitor, the necrosis elicitor PcCRN20-624 was co-infiltrated with RiCRN1:eGFP, RiCRN1ΔSP:eGFP and RiCRN1-C:eGFP. Co-infiltration of PcCRN1_719 with the three RiCRN1 constructs as well as PcCRN20_624 with eGFP served as negative controls. Photographs were taken 7 days after infiltration. Quantification is shown as numbers of leaves displaying necrosis compared to the total amount of leaves assayed per construct. Cell death induced upon PsCRN20_624 was never inhibited by RiCRN1 versions.

As mentioned above, CRN proteins were initially described as triggers for crinkling and necrosis phenotypes *in planta* (Torto et al., 2003). However, further functional analyses of this large family showed that although some CRNs do induce necrosis, many others do not, and some of them rather function by preventing host cell death (Liu et al., 2011; Shen et al., 2013; Rajput et al., 2014, 2015). Thus, one of the functions ascribed to several CRNs is to be regulators of cell death (Amaro et al., 2017). In order to investigate the ability of RiCRN1 to induce cell death, we infiltrated *N. benthamiana* leaves with *Agrobacterium tumefaciens* carrying RiCRN1 fused to GFP expressed under the control of the CaMV 35 S promoter. The assay was performed either with the full-length sequence (RiCRN1:eGFP), the carboxy terminus (RiCRN1-C:eGFP) or with RiCRN1ΔSP:eGFP lacking the signal peptide. As positive and negative controls, the *Phytophthora capsici* effectors PcCRN20_624 and PcCRN1_719 were used (Stam et al., 2013a,b), both also fused to GFP and under the control of the CaMV 35S promoter. The results showed that none of the RiCRN1 versions are able to induce cell death in *N. benthamiana* leaves, similar to the negative control PcCRN1_719 or to GFP. In contrast, leaf areas infiltrated with the positive control, the effector PcCRN20_624, clearly showed cell death symptoms (**Figure 6A** and **Supplementary Figure 4**). To test whether RiCRN1 could prevent the cell death elicited by PcCRN20_624, co-infiltration experiments were carried out. However, none of the RiCRN1 versions were able to decrease the number of cell death events elicited by PcCRN20_624 (**Figure 6B**). These results suggest that the function of RiCRN1 is not associated to cell death processes, at least in leaves. This is perhaps not surprising given that *R. irregularis* only colonizes roots and not leaves. However, expression of *RiCRN1* in *M. truncatula* roots (see below) or in *A. thaliana* plants (results not shown) does not induce cell death. Thus, it indicates that RiCRN1 function is not related to cell death but rather to the colonization process of the fungus, as the expression analysis suggests.

### RiCRN1 Silencing by HIGS Leads to a Reduction in Mycorrhizal Colonization

**FIGURE 7:**
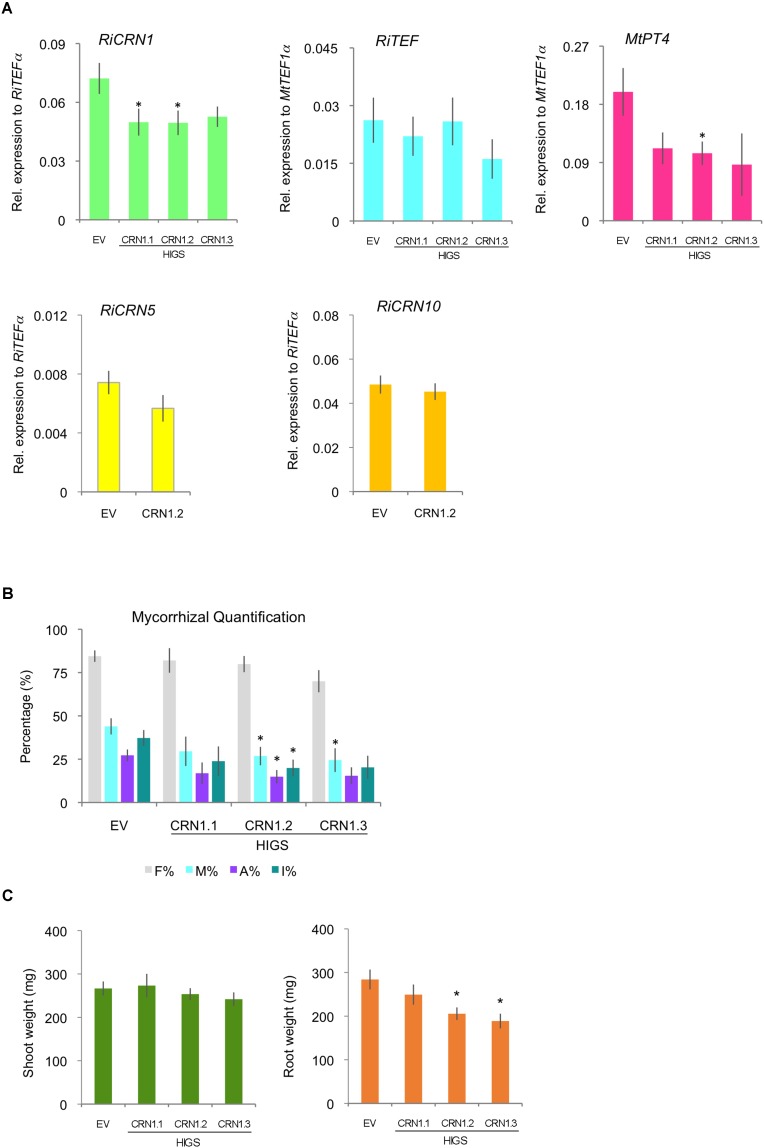
Host-induced gene silencing of *RiCRN1* in mycorrhizal *M. truncatula* plants. **(A)** *M. truncatula* composite plants expressing three RNAi constructs targeting different regions of the gene *RiCRN1* (HIGS CRN1.1, CRN1.2, and CRN1.3) were inoculated with *R. irregularis*. Expression of *RiTEF1α*, *MtPT4* and the *R. irregularis* effectors *CRN1*, *CRN5*, and *CRN10* were analyzed at 5 wpi. Expression is shown as relative expression to *MtTEF1α* for *MtPT4* and *RiTEF1α* or to *RiTEF1α* for *RiCRN1*, *RiCRN5*, and *RiCRN10. RiCRN1* is downregulated compared to the EV control, significantly for constructs HIGS CRN1.1 and HIGS CRN1.2. In contrast, *RiCRN5* and *RiCRN10* as potential off-targets are not. The expression of the arbuscule marker *MtPT4* is reduced in plants expressing all three constructs, being significant for HIGS CRN1.2. Expression of *RiTEF1α* is not changed as compared to the EV control. Error bars represent SEM with *n* = 9 biological replicates for each condition. Student’s *t*-test was used to validate significance of expression between the three different RNAi conditions and the EV control (^∗^*p* ≤ 0.05). **(B)** Mycorrhizal quantification was calculated after Trouvelot et al. (1986). Intensity of colonization (M%), arbuscule abundance (A%), and hyphal abundance (I%) in the root system are significantly reduced in HIGS CRN1.2 compared to the EV control. M% is also significantly reduced in HIGS CRN1.3. While frequency of colonization in the root system (F%) is constant in all conditions. Error bars represent SEM with *n* = 9 biological replicates for each condition. Student’s *t*-test was used to validate significance of results comparing the different RNAi conditions with the EV plants (^∗^*p* ≤ 0.05). **(C)** Shoot and root fresh weight was also determined at 5 wpi. Compared to the EV control, none of the *RiCRN1* HIGS constructs seem to have an effect on shoot weight. In contrast, HIGS CRN1.2 and HIGS CRN1.3 induced a significant reduction in root biomass.

In order to link the function of RiCRN1 to the colonization of the fungus and the establishment of the symbiosis, and given that AM fungi cannot be genetically manipulated, we attempted its inactivation using HIGS (Helber et al., 2011). There has been, as far as we know, few publications where CRN proteins were inactivated in other organisms, possibly given the difficulty to transform them. However, Liu et al. (2011) successfully silenced *PsCRN63* and *PsCRN115* in *P. sojae* and interestingly those silenced lines had a reduced virulence that correlated with a diminished ability to suppress cell death and defense responses. Also, silencing *PcCRN4* in *P. capsici* reduced the pathogen virulence and boosted the host defenses of the plant (Mafurah et al., 2015). To silence *RiCRN1*, three silencing constructs were employed that targeted different regions of the gene and nine transgenic plants were used per construct. Plants were then mycorrhized for 5 weeks. The results showed that two of the constructs did significantly downregulate the expression of *RiCRN1 in planta* (**Figure 7A**), albeit only ca. 30%, as compared to control plants transformed with an empty vector (EV). This is not surprising considering the mechanism of function of HIGS and the difficulties of obtaining fully downregulated fungal transcripts with this methodology. Nevertheless, the moderate downregulation led to a decrease in mycorrhizal intensity (M%) for two of the constructs and in one of them, HIGS-CRN1.2, also to a significant reduction in the number of arbuscules (A%) (**Figure 7B**). This correlated with a significantly lower expression of the arbuscule specific marker, the symbiotic plant phosphate transporter *MtPT4* (**Figure 7A**) in these silenced plants. In addition, mycorrhization of HIGS-CRN1 plants had an impact on root biomass, with a significant decrease observed in HIGS-CRN1.2 and HIGS-CRN1.3 plants (**Figure 7C**). Given that RiCRN1 is highly homologous to RiCRN5 and RiCRN10 that are also expressed *in planta*, we analyzed the off-target effects of the HIGS-CRN1.2 silencing construct on these two CRNs (**Figure 7A**). The results showed that HIGS-CRN1.2 did not silence *RiCRN5* or *RiCRN10*. Altogether, these data indicate that reduction of *RiCRN1* expression affects symbiosis progression and plant growth.

### Ectopic Expression of *RiCRN1* Impacts on Arbuscule Development

**FIGURE 8:**
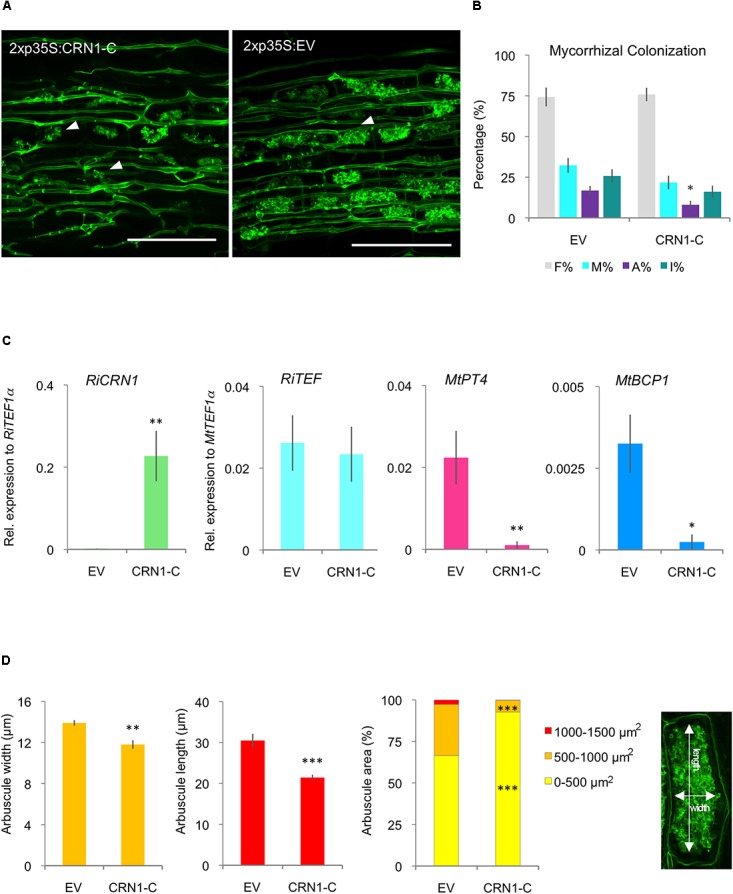
Ectopic expression of *RiCRN1* in mycorrhizal *M. truncatula* plants. **(A)** *M. truncatula* composite plants expressing *RiCRN1-C* were inoculated with *R. irregularis*. Visualization of *R. irregularis* colonization at 5 wpi was carried out by wheat-germ agglutinin (WGA)-FITC immunostaining. Arrowheads indicate arbuscules in both conditions. Note the smaller arbuscule size in *RiCRN1-C* overexpressing plants. Scale bars represent 100 μm. **(B)** Mycorrhizal quantification was made after Trouvelot et al. (1986). A reduction of arbuscule abundance in the root system (A%) is significant compared to the EV control. Error bars represent SEM with *n* = 6 biological replicates for each condition. Student’s *t*-test was used to calculate significance of results, comparing *RiCRN1-C* expressing with EV plants (^∗^*p* ≤ 0.05). **(C)** Expression of *RiTEF1α*, *MtPT4*, *MtBCP1*, and the *R. irregularis* effector *CRN1* were analyzed 5 wpi. Expression is shown as relative expression to *MtTEF1α* for *MtPT4*, *MtBCP1*, and *RiTEF1α* or to *RiTEF1α* for *RiCRN1*. Error bars represent SEM with *n* = 6 biological replicates for each condition. Student’s *t*-test was used to calculate significance, comparing *RiCRN1* expressing with EV plants (^∗^*p* ≤ 0.05). **(D)** Arbuscule size and distribution in arbuscule populations of mycorrhizal *M. truncatula* roots 5 wpi. Arbuscules were measured in width and length as illustrated. Distribution of arbuscule size (area) within the root system is shown in percentage. Arbuscules were categorized based on area (0–500, 500–1000, and 1000–1500 μm^2^). Data represent averages of *n* = 6 biological replicates. About 8–10 randomly selected infection sites were used to measure *n* ≥ 200 arbuscules in each biological replicate. Error bars represent SEM. Student’s *t*-test was used to calculate significance between *RiCRN1-C* and EV composite plants (^∗^*p* ≤ 0.05; ^∗∗^*p* ≤ 0.01; ^∗∗∗^*p* ≤ 0.001).

To further get insights into the specific function of RiCRN1 during symbiosis, transgenic plants ectopically expressing the effector domain (RiCRN1-C) were created. It has been shown for some CRN effectors such as AeCRN13 from *A. euteiches* or PcCRN4 from *P. capsici*, that their expression *in planta* increases the susceptibility towards the pathogenic oomycete *Phytophthora capsici* (Mafurah et al., 2015; Ramirez-Garces et al., 2016). Thus, we hypothesized that the expression of *RiCRN1*, which increases concomitantly to the expression of *MtPT4*, could be related to a mechanism to facilitate the development of arbuscules. The expression of *RiCRN1-C* did not significantly modify plant growth nor produced browning symptoms that could indicate cell death induction (**Supplementary Figure 5**). This is different from the results observed in *M. truncatula* plants ectopically expressing *CRN13* from *A. euteiches* and *B. dendrobatidis*, that were abnormally developed (Ramirez-Garces et al., 2016). Also, expression of *RiCRN1-C* did not affect the mycorrhizal colonization levels (**Figures 8A,B**). However, in contrast to our prediction, the number of arbuscules was significantly reduced (**Figure 8B**). In agreement, overexpression of *RiCRN1-C* did not alter the *RiTEF1α* levels in roots but almost abolished the expression of the phosphate transporter *MtPT4* (**Figure 8C**). Because silencing of *RiCRN1* also impacted in arbuscule function, we wondered whether the negative effect of the ectopic expression of *RiCRN1* on arbuscule number was related to the development of new arbuscules. Thus, we also analyzed the expression of *MtBCP1*, a marker for arbuscule initiation (Gutjahr and Parniske, 2013), and similarly to *MtPT4*, its expression was much reduced in roots overexpressing *RiCRN1-C* (**Figure 8C**). These results suggest that RiCRN1 plays a specific role during arbuscule development. Morphometric analyses of arbuscules in *RiCRN1-C* overexpressing roots confirmed that hypothesis, showing that arbuscules were not only less but also significantly smaller than in control plants (**Figures 8A,D**). Furthermore, the size distribution of arbuscules between both treatments was significantly altered, with a much higher proportion of smaller arbuscules (0–500 μm^2^) in RiCRN1-C roots and a reduction in larger arbuscules (**Figure 8D**). Thus, it appears that RiCRN1 rather plays a role in controlling arbuscule initiation, and that the amount of transcript together with the time and place of expression is critical for its proper function. Arbuscule development is a highly concerted process where many genes have to act in a coordinated manner (Gutjahr and Parniske, 2013; MacLean et al., 2017). In this scenario, *RiCRN1* accumulates during mycorrhiza establishment parallel to arbuscule development suggesting that it is mainly expressed in arbuscules, and that its targets might be located in plant cells that harbor them. In contrast, overexpression of *RiCRN1* is constitutive, high, and ubiquitous and therefore it might prevent the coordinated action on its plant targets.

## Conclusion

Here, we show that AM fungi contain a repertoire of putative CRN effectors that besides the SP7-like proteins represent a new family of effectors characterized in an AM fungus. This is unique because although CRN effectors are widespread in plant-pathogenic oomycetes, they have never been described in a fungus interacting with plants. Most of the AM CRNs do not contain a signal peptide and thus, if they are secreted, the mechanisms of their unconventional secretion should be investigated. A small set of *R. irregularis* predicted CRNs contains a canonical signal peptide, and several of them accumulate during fungal development *in planta*. Here, we have functionally analyzed RiCRN1 and showed that it localizes in nuclear bodies different from speckles, forms dimers and does not induce plant cell death. Its expression pattern and its effect during symbiosis suggest an involvement in arbuscule development. Thus, *RiCRN1* expression is necessary for symbiosis progression as shown by the silencing experiments, and its ectopic expression negatively impacts the initiation of arbuscule formation. However, given that several other similar CRNs exist and are expressed during symbiosis, more studies of the whole family and their plant targets will be required to fully understand the role of *R. irregularis* CRNs in the AM symbiosis.

## Author Contributions

SV and NR conceived the experiments and wrote the manuscript. SV, RB, and SH carried out all the experimental works. NC provided the initial sequence information.

## Conflict of Interest Statement

The authors declare that the research was conducted in the absence of any commercial or financial relationships that could be construed as a potential conflict of interest.

## Footnotes

1 http://www.cbs.dtu.dk/services/SignalP-3.0/

2 http://www.cbs.dtu.dk/services/SignalP/

3 https://psort.hgc.jp/form2.html

4 http://www.ebi.ac.uk/Tools/msa/clustalo/

5 http://www.jalview.org/node/13

6 http://weblogo.berkeley.edu

7 http://www.sbg.bio.ic.ac.uk/phyre2/

8 https://www.rcsb.org/

9 http://fiji.sc/Fiji
