# Supplementary material for: RiCRN1, a Crinkler Effector From the Arbuscular Mycorrhizal Fungus Rhizophagus irregularis, Functions in Arbuscule Development
Source: Front Microbiol. 2018 Sep 4;9:2068. doi: 10.3389/fmicb.2018.02068 (PMC6131194; doi:10.3389/fmicb.2018.02068)
Supplement: Supplementary file 4 [file Data_Sheet_1.PDF]

Supplementary Figure 1

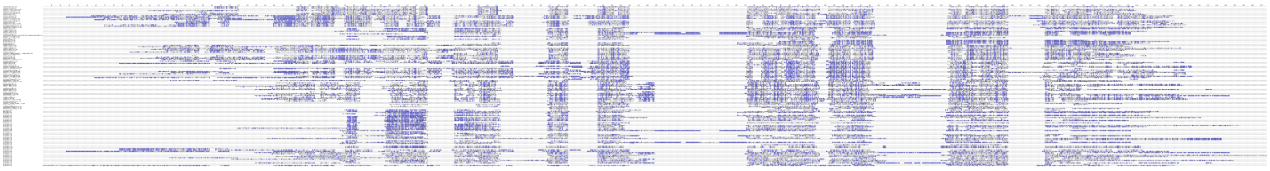

Supplementary Figure 1. Global alignment of RiCRN proteins from different genome data sets. 82 (Tisserant *et al*., 2013; JGI) and 43 (Lin *et al*., 2014) RiCRN sequences (see Supplementary Table 1) were aligned using ClustalO (BLOSUM62). This revealed the clustering of several members into groups with highly conserved N- and C-terminal amino acid stretches.

Supplementary Figure 2

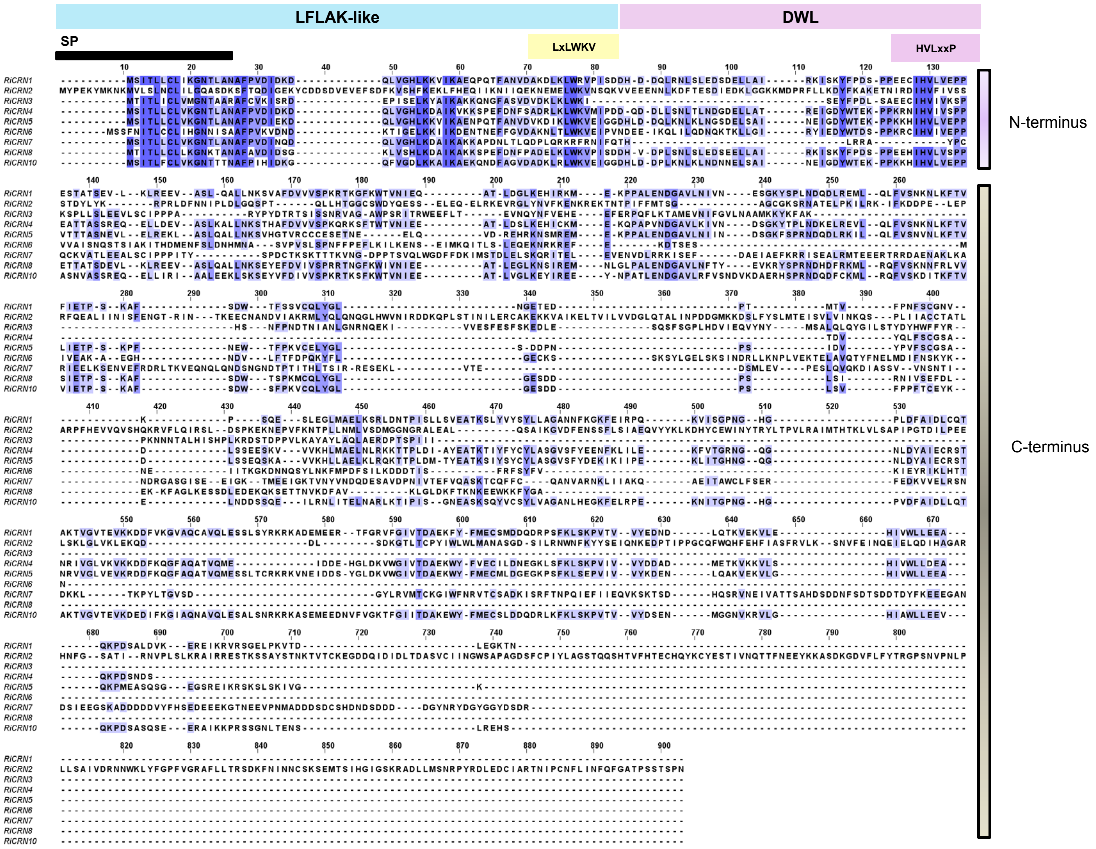

Supplementary Figure 2. Alignment of all RiCRN members with predicted secretion signal. A highly conserved N-terminal region, defined by the *Phythophtora* characteristic terminal HVLxxP motif, is followed by more diverse C-terminal sequences. Alignment was performed using ClustalO. Identical amino acids are highlighted in blue.

Supplementary Figure 3

(A)

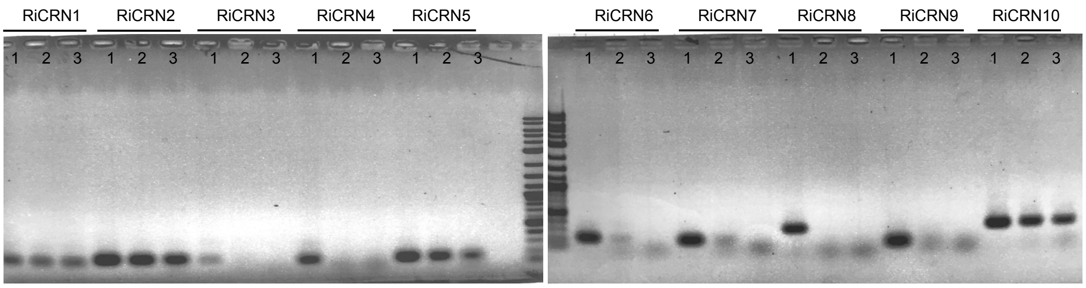

(B)

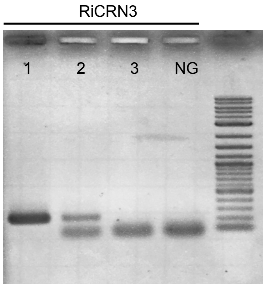

Supplementary Figure 3. Expression of *R. irregularis CRNs* containing a signal peptide. (A) The expression of *RiCRN1* to *8* and *RiCRN10* was analyzed by conventional PCR in cDNA from *R. irregularis* spores (2) or from *M. truncatula* mycorrhizal roots (3). Presence in the genome was verified by PCR on genomic DNA from *R. irregularis* (1). Expression in mycorrhizal roots was confirmed for *RiCRN1, RiCRN2, RiCRN5* and *RiCRN10* **(B)** While PCR oligonucleotides for *RiCRN3* failed to produce an amplicon from *R. irregularis* genomic DNA (Supplementary Figure 3A), a new set of PCR oligonucleotides was able to amplify *RiCRN3* from gDNA as well as from cDNA generated from spores.

Supplementary Figure 4

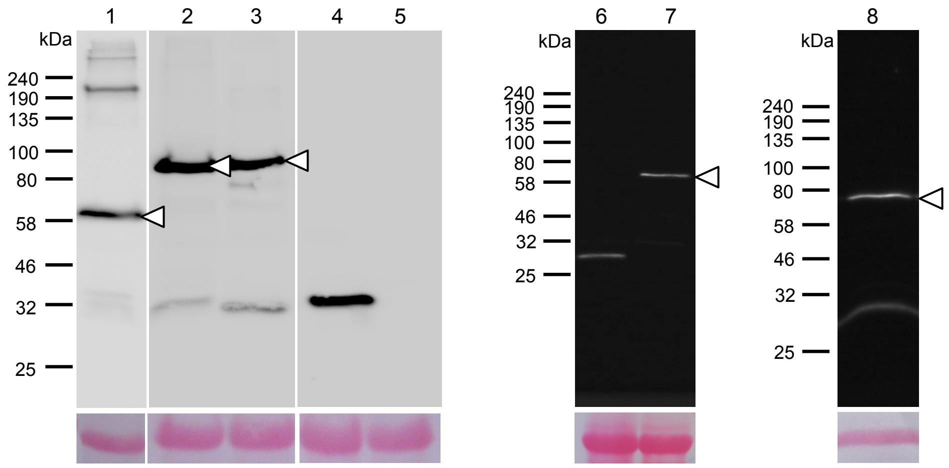

1 PcCRN20_624	58.4 kDa
2 PcCRN1 _719	81.5 kDa
3 RiCRN1ΔSP	79.7 kDa
4 eGFP	26.9kDa
5 WT	
6 eGFP	26.9 kDa
7 RiCRN1-C	78.0 kDa
8 RiCRN1	81.3 kDa

Supplementary Figure 4. Confirmation of integrity of RiCRN1-GFP fusion proteins in *N. benthamiana.* To analyze protein integrity of RiCRN1:eGFP (81.3 kDa), RiCRN1ΔSP:eGFP (79.7 kDa), RiCRN-C:eGFP (78 kDa), eGFP:PcCRN20_624 (58.4 kDa) and eGFP:PcCRN1_719 (81.5 kDa), a western blot analysis was employed. Protein extract of *N. benthamiana* ground leaf tissue, expressing these constructs as well as a eGFP and a wildtype control were separated in a SDS-PAGE and subsequently blotted on nitrocellulose membrane. Ponceau S staining shows successful transfer of the protein to the membrane. Anti-GFP primary antibodies (rabbit) were paired with horseradish peroxidase-coupled anti-rabbit antibodies (goat) for chemiluminescence immuno-detection. Fusion proteins are indicated with arrowheads. In all lanes free eGFP migrates at 27 kDa due to cleavage in the process of protein extraction.

Supplementary Figure 5

(A)

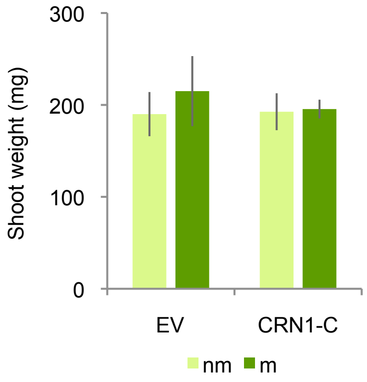

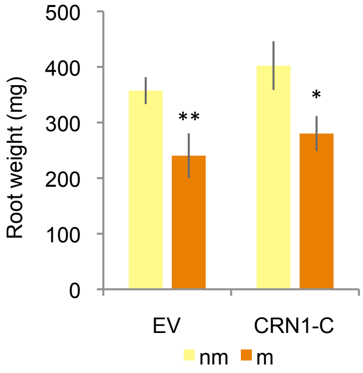

(B)

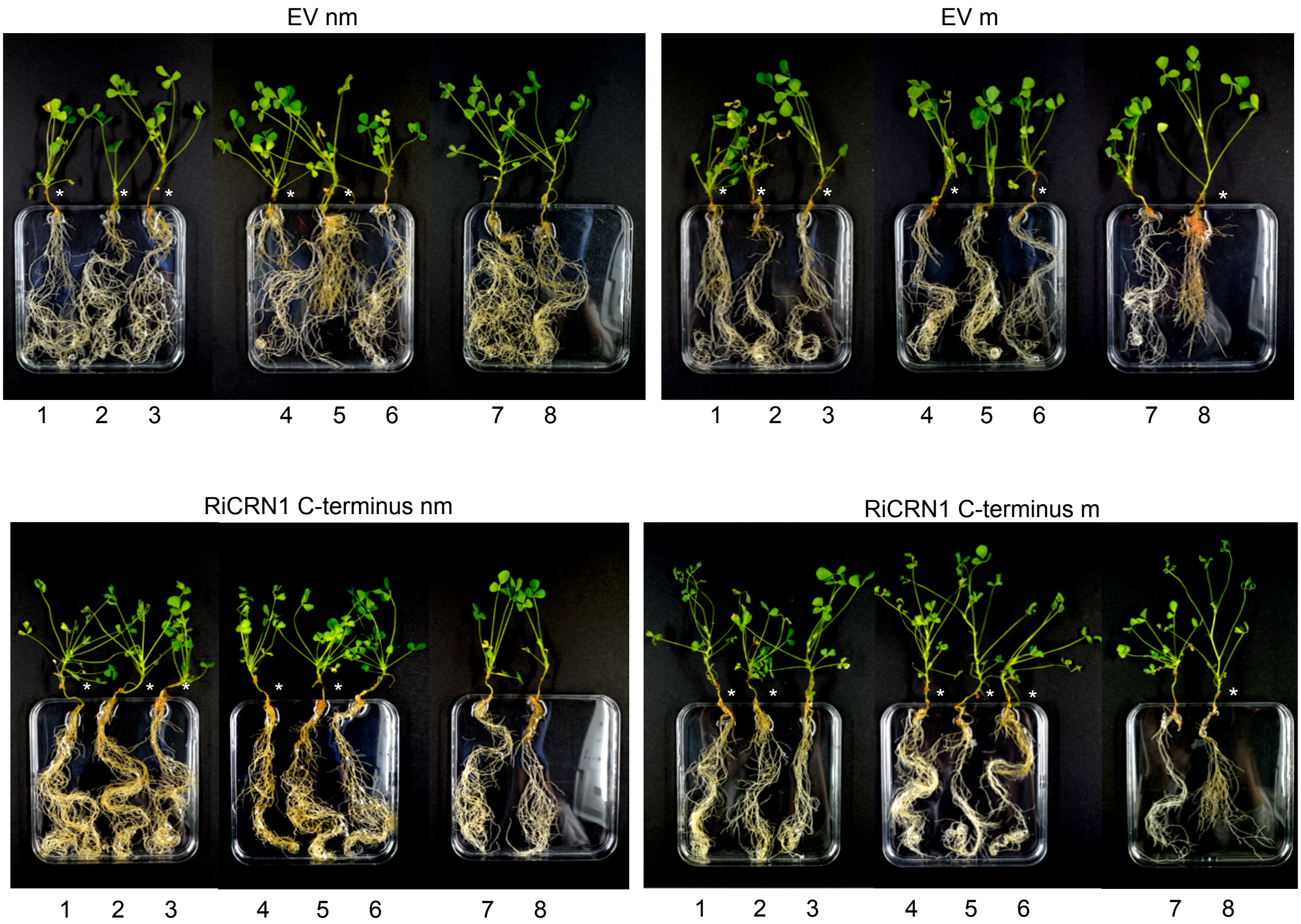

Supplementary Figure 5. Morphological evaluation of *M. truncatula* composite plants expressing *RiCRN1-C.* **(A)** *M. truncatula* composite plants expressing *RiCRN1-C* and the EV control were inoculated with *R. irregularis* (m) or not (nm). Shoot and root fresh weight was measured 5 wpi. No difference in shoot fresh weight is seen between all conditions. Root biomass shows significant reduction in mycorrhizal plants in *RiCRN1-C* and EV plants, while root fresh weight does not change between *RiCRN1-C* and control plants. Error bars represent standard error of the mean with n=5 biological replicates for non-mycorrhizal plants and n=6 biological replicates for mycorrhizal plants. Student's t-test was used to validate significance with ($p^* \leq 0.05$ and $p^{**} \leq 0.01$). **(B)** Photographs of *M. truncatula* composite plants expressing *RiCRN1-C,* 5 wpi. Asterisks mark biological replicates analyzed further, based on fluorescence intensity of the integrated DsRED cassette.
